# Reevaluating the nature of thymic lipofibroadenoma: A case report with hamartomatous features and a literature review

**DOI:** 10.1097/MD.0000000000048875

**Published:** 2026-05-29

**Authors:** Ning Zhou, Shuya Hu, Liqiao Chen, Hegang Wu, Fanrong Wang

**Affiliations:** a Department of Pathology, Mianyang 404 Hospital, Mianyang, Sichuan, China; b Department of Pathology, The First People’s Hospital of Yibin, Yibin, Sichuan, China.

**Keywords:** hamartoma, lipofibroadenoma, thymic tumor, thymoma

## Abstract

**Rationale:**

Thymic lipofibroadenoma (LFA) is an extremely rare benign thymic tumor of mixed epithelial and mesenchymal origin, whose pathological nature remains incompletely understood. This case presents a unique thymic lesion combining classic LFA features with a novel lobulated, ductal-forming epithelial proliferation, prompting a reevaluation of its classification.

**Patient concerns:**

A 68-year-old male nonsmoker with a 10-year history of hypertension was admitted following a motor vehicle accident. Chest computed tomography incidentally revealed a well-circumscribed, heterogeneous mass (55 × 38 × 65 mm) in the right anterior mediastinum, containing both soft-tissue and adipose components. The patient had no specific symptoms related to the mass.

**Diagnoses:**

Histological examination showed a biphasic architecture: areas resembling classic thymic LFA with anastomosing bland epithelial strands within a fibroadipose stroma, and a novel component with lobulated hyperplastic epithelium exhibiting ductal differentiation and bilayered glandular structures. Immunohistochemically, epithelial cells were diffusely positive for CK5/6 and p63; ductal structures showed a bilayered pattern (CK7+/p63− luminal cells; p63+ basal cells). The Ki-67 proliferation index was low (<2%). No cytologic atypia, mitosis, or necrosis was seen. Based on the composite hamartomatous morphology, the lesion was diagnosed as a thymic hamartoma.

**Interventions:**

The tumor was completely excised via a transxiphoid single-port thoracoscopic approach. The postoperative recovery was uneventful.

**Outcomes:**

At 21-month follow-up, the patient remained disease-free with no evidence of recurrence or residual disease on radiographic imaging.

**Lessons:**

This unique thymic lesion, featuring LFA-like areas admixed with duct-forming lobulated epithelial proliferation, is best classified as a thymic hamartoma. Review of the literature suggests that previously reported LFAs may also represent hamartomatous lesions rather than true neoplasms. Complete surgical resection appears curative. Further case accumulation and molecular studies are needed to elucidate the pathogenesis.

## 1. Introduction

Thymic neoplasms are predominantly thymomas. According to the 2021 World Health Organization (WHO) Classification of Thoracic Tumors, thymic epithelial tumors are classified based on their cytologic and histologic features. Within this framework, thymomas with benign cytology include types A, AB, B1, B2, and B3, as well as micronodular thymoma with lymphoid stroma, metaplastic thymoma, and lipofibroadenoma (LFA).^[1]^ With the exception of LFA, all thymoma subtypes possess malignant potential. LFA is an extremely rare mixed epithelial and mesenchymal tumor of the thymus. To date, only a limited number of cases have been reported in the English-language literature, and its pathologic characteristics and biological behavior remain incompletely understood. Herein, we report a distinctive thymic lesion composed of a disorganized admixture of classical LFA-like areas and lobulated hyperplastic epithelium exhibiting ductal differentiation. To the best of our knowledge, this combination of histologic features has not been previously reported. This report details the clinical and pathologic findings of this novel entity.

## 2. Materials and methods

### 2.1. Clinical presentation

A 68-year-old male nonsmoker was hospitalized following a motor vehicle accident. His medical history was notable for hypertension of 10 years’ duration, with no other significant comorbidities or remarkable family history. Physical examination revealed no obvious abnormalities, and all blood biochemistry parameters were within normal limits. Chest computed tomography revealed a well-circumscribed, heterogeneous mass in the right anterior mediastinum, measuring approximately 55 × 38 × 65 mm, which contained both soft-tissue and adipose components. No mediastinal lymphadenopathy or pleural effusion was observed. Based on the imaging features, the differential diagnosis included thymoma and teratoma. The patient underwent complete tumor resection via a transxiphoid single-port thoracoscopic approach, and his postoperative recovery was uneventful.

### 2.2. Histological assessment

All tissue specimens were fixed in 10% formalin and routinely processed for paraffin embedding. Sections (3–4 μm) were cut and stained with hematoxylin and eosin for light microscopic evaluation. Immunohistochemical studies and in situ hybridization were performed according to standard diagnostic protocols.

### 2.3. Immunohistochemistry

All antibodies (prediluted) and kits were purchased from Zhongshan Golden Bridge Biotechnology Company and used in accordance with the manufacturer’s instructions. Immunohistochemical staining was performed using a Dako automated immunohistochemistry instrument (Agilent). All immunostaining procedures were carried out as described previously, with appropriate positive and negative controls included. The primary antibodies used in this study are listed in Table 1.

**Table 1:** Performed immunohistochemical stains, with interpretation and technical data.

Antibody	Manufacturer	Species	Clone	Dilution	Stainer
CK5/6	ZSB	mouse	OTI1C7	predilute	Dako Autostainer Link 48
P63	ZSB	rabbit	B18	predilute	Dako Autostainer Link 48
CK7	ZSB	mouse	UMAB161	predilute	Dako Autostainer Link 48
CD3	ZSB	mouse	LN10	predilute	Dako Autostainer Link 48
CD20	ZSB	mouse	L26	predilute	Dako Autostainer Link 48
TdT	ZSB	rabbit	EP266	predilute	Dako Autostainer Link 48
Ki-67	ZSB	mouse	MIB-1	predilute	Dako Autostainer Link 48

CD = cluster of differentiation, CK = cytokeratin, Ki-67 = antigen identified by monoclonal antibody, TdT = terminal deoxynucleotidyl transferase, ZSB = Beijing Zhongshan Golden Bridge Biotechnology Company (Beijing, China).

## 3. Results

### 3.1. Gross and microscopic findings

**Figure 1.**
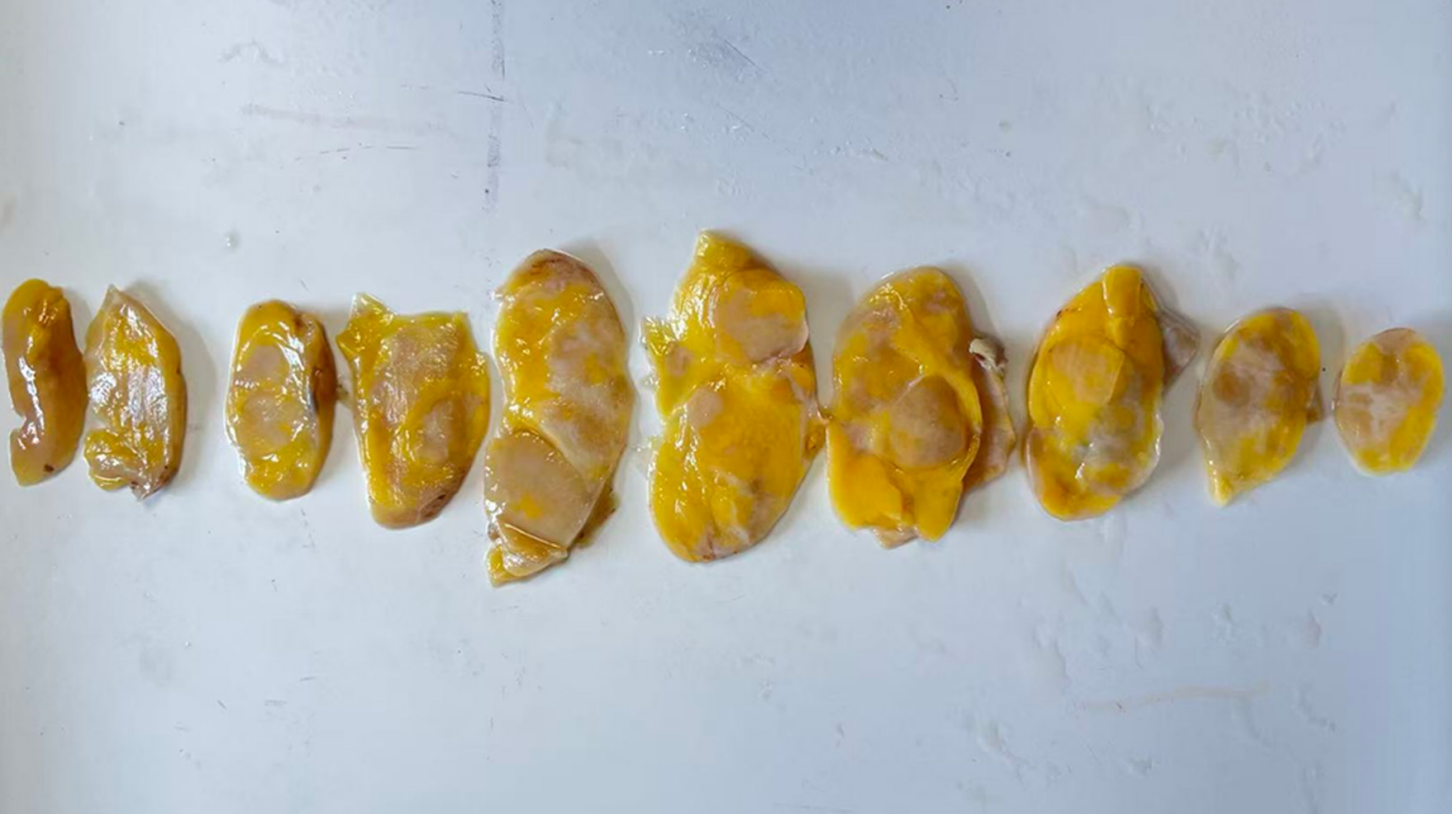
Macroscopically, the tumor’s cut surface exhibited an appearance characterized by alternating yellow and white areas.

**Figure 2.**
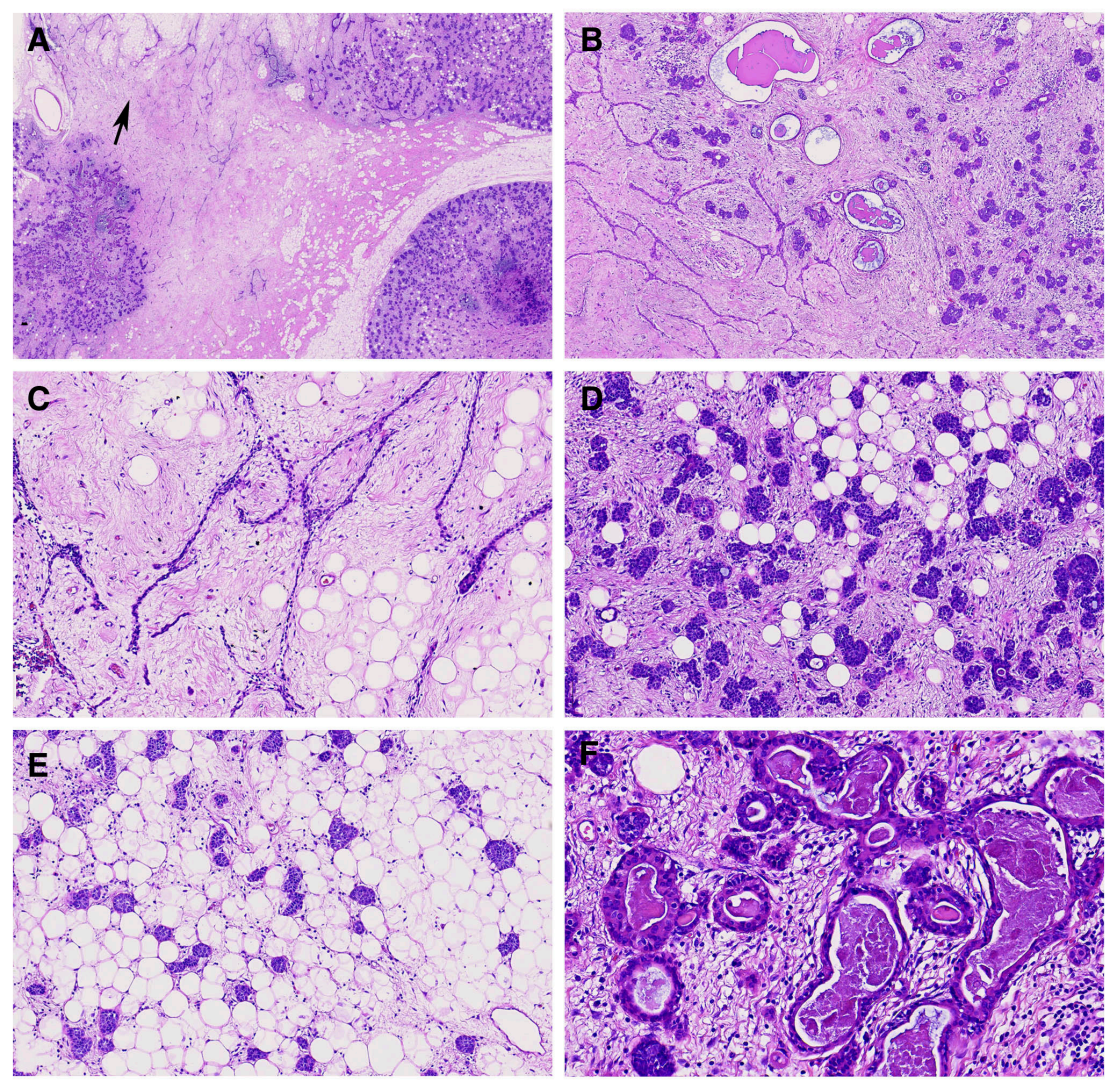
(A) Lobular hyperplastic epithelium admixed with classical LFA-like areas (arrow) (× 40); (B) The 2 components were haphazardly intermingled, with focal ductal differentiation (× 100); (C) The LFA-like areas showed anastomosing strands of bland epithelium within a loose fibrous stroma that contained adipocytes (× 200); (D), (E) Uniform epithelial nests distributed within a fibroadipose background, resembling a mammary hamartoma (× 200); (F) Bilayered ductal structures (× 400). LFA = lipofibroadenoma.

Macroscopically, the resected specimen was well-encapsulated and clearly demarcated from the surrounding thymic tissue. The cut surface exhibited a heterogeneous appearance with alternating yellow and white areas(Fig. 1). Histologically, the tumor showed a biphasic architecture (Fig. 2A and 2B). The first component resembled classic thymic LFA, comprising narrow, anastomosing strands of bland thymic epithelial cells within a loose fibrous stroma interspersed with mature adipocytes (Fig. 2C): an architectural pattern reminiscent of a breast fibroadenoma. The second component resembled a mammary hamartoma and was characterized by proliferative epithelial cells arranged in a lobulated pattern, mixed with adipose tissue and fibrous stroma in varying proportions (Fig. 2A, 2D, and 2E). Focal areas displayed ductal differentiation, evidenced by bilayered glandular structures (Fig. 2B and 2F). The 2 components were haphazardly intermingled throughout the lesion (Fig. 2B). Epithelial cells exhibited no significant cytologic atypia, mitotic activity, or necrosis. Occasional small lymphoid aggregates containing Hassall corpuscles were entrapped within the stromal component.

### 3.2. Immunohistochemistry

**Figure 3.**
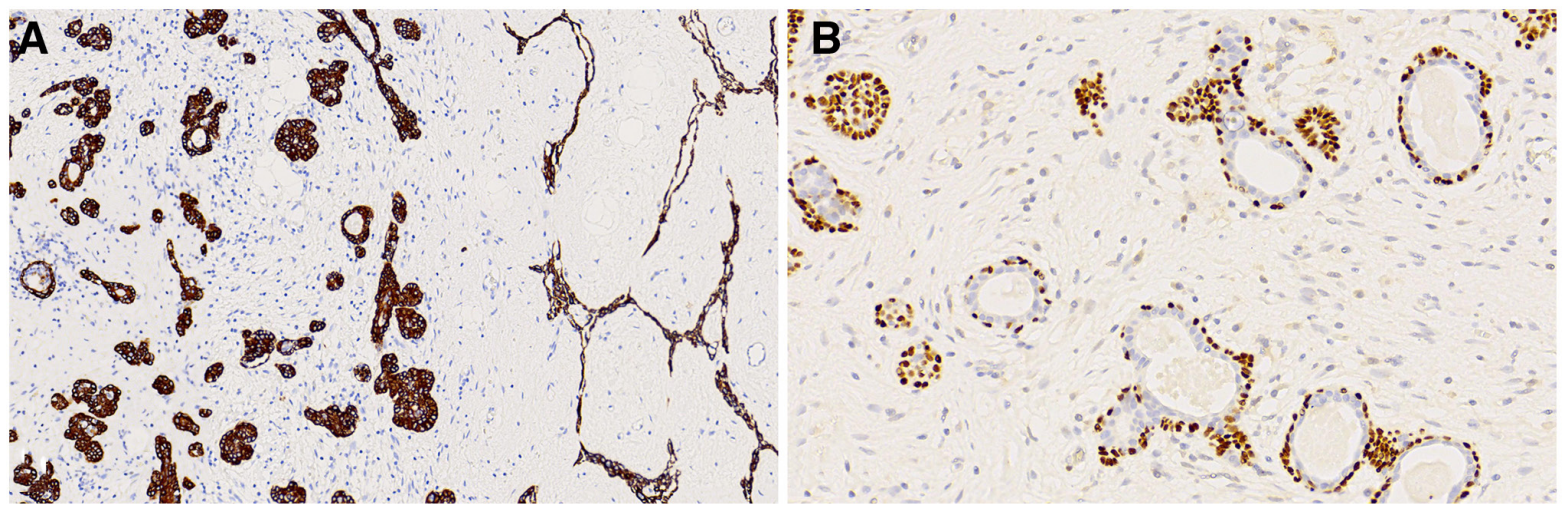
(A) Both the epithelial strands and the lobular hyperplastic epithelium were diffusely positive for CK5/6 (× 200). (B) The outer layer of ductal cells was positive for p63, while the inner luminal cells were negative (× 400). CK = cytokeratin.

Immunohistochemical analysis demonstrated that both the epithelial strands and the lobular hyperplastic epithelium were diffusely positive for cytokeratin (CK)5/6 (Fig. 3A) and p63. CK7 expression was strong in the lobular and ductal components but weak to absent in the strands. Within the ductal structures, p63 staining was restricted to the outer cell layer, with the inner luminal cells being negative (Fig. 3B). Background lymphocytes expressed cluster of differentiation (CD)3 and CD20, with only terminal deoxynucleotidyl transferase (TdT)-positive immature T lymphocytes identified within the residual thymic tissue incorporated into the tumor. The antigen identified by monoclonal antibody proliferation index was low (< 2%) in both components.

### 3.3. Pathological diagnosis and treatment

Given the unique combination of histologic features (including a benign epithelial proliferation, adipose tissue, fibrous connective tissue, ductal differentiation, and the absence of significant atypia or proliferative activity) along with the immunophenotypic profile, this lesion did not conform completely to any established category of thymic tumor. Based on its hamartomatous morphology, we propose the diagnostic term “thymic hamartoma” for this entity. The patient recovered well postoperatively and showed no evidence of recurrence or residual disease on radiographic follow-up 21 months after surgery.

## 4. Discussion

The thymus is a small, encapsulated lymphoid organ situated in the anterior mediastinum. It comprises epithelial cells forming the outer cortex, along with myeloid cells and lymphocytes located in the germinal centers. As the primary site of T-cell maturation, the thymus plays a critical role in adaptive immunity. Although primary thymic tumors are uncommon, the most frequent histologic subtype is thymoma: a neoplasm derived from thymic epithelial cells, which are essential for orchestrating T-cell development. The fifth edition of the WHO Classification of Thoracic Tumours (2021) introduces significant updates to the taxonomy of thymic epithelial neoplasms, refining diagnostic criteria through the integration of morphologic, immunophenotypic, and molecular-genetic features.^[1]^ This edition maintains a broad division into 3 major categories (thymomas, thymic carcinomas, and thymic neuroendocrine tumors) and emphasizes the prognostic and biologic importance of precise histologic subtyping.

Thymoma is an uncommon neoplasm originating from the epithelial cells of the thymus, typically located in the anterior mediastinum. Its incidence ranges from 0.13 to 0.26 cases per 100,000 person-years, accounting for approximately 50% of all anterior mediastinal tumors.^[2]^ However, it constitutes < 0.5% of all adult malignancies.^[2]^ The clinical presentation of thymoma varies widely. Many patients are asymptomatic at diagnosis, with the tumor discovered incidentally during imaging performed for unrelated indications. Symptomatic patients may present with chest pain, discomfort, cough, or dyspnea. A hallmark of thymoma is its frequent association with paraneoplastic syndromes and autoimmune disorders, including myasthenia gravis, polymyositis, Lambert–Eaton myasthenic syndrome, acquired neuromyotonia, Morvan syndrome, encephalitis, and pure red cell aplasia.^[3]^ Myasthenia gravis is the most frequently associated condition; studies indicate that 15 to 20% of patients with myasthenia gravis have a thymoma, while approximately 30% of thymoma patients develop myasthenia gravis.^[3,4]^

Thymomas are classified into several subtypes, including type A (with an atypical variant), type AB, type B (subdivided into B1, B2, and B3), micronodular thymoma with lymphoid stroma, metaplastic thymoma, and LFA. This classification is based primarily on the morphology of the epithelial cells (spindle/oval vs dendritic/epithelioid) and the relative proportion of immature T lymphocytes to neoplastic epithelial cells. Type A thymoma consists of bland spindle or oval epithelial cells with few or no admixed TdT-positive immature T lymphocytes. Type AB thymoma is characterized by a biphasic pattern, combining type A-like areas with lymphocyte-rich regions resembling type B thymoma. Type B thymomas are further classified into B1, B2, and B3, reflecting an increasing epithelial-to-lymphocyte ratio and higher degrees of cytologic atypia. Type B3 thymoma exhibits more pronounced epithelial atypia but is distinguished from thymic carcinoma by the absence of overtly malignant cytologic features and a generally less aggressive growth pattern, although it may display capsular invasion. These refinements facilitate more accurate diagnosis and improved clinical management of these uncommon tumors.

Thymic LFA is a rare thymic tumor of uncertain origin. Its clinical and pathologic characteristics have not been fully elucidated due to its extreme rarity.^[5]^ This entity was first defined by Kuo et al in 2001.^[6]^ To date, only a handful of cases have been reported in the English-language literature; their clinical features are summarized in Table 2. Throughout the literature (including the present case) these tumors exhibit remarkably consistent characteristics. They almost invariably present as well-demarcated masses in the anterior mediastinum; frank invasion of adjacent mediastinal adipose tissue has been reported only once.^[7]^ The reported cohort comprises 8 males and 6 females, with a wide age range at presentation (17–62 years) and a median age of 35.6 years, suggesting a predisposition toward younger adults. Tumor sizes vary considerably, ranging from 3.9 cm to 23 cm in greatest dimension (mean approximately 10 cm; size was not reported in 1 case). In this series, patients most commonly presented with localized, nonspecific symptoms such as cough, dyspnea, or chest pain, although many cases were discovered incidentally. Two of the 14 documented cases occurred in association with a type B1 thymoma,^[6,10]^ one of these was also associated with pure red cell aplasia.^[6]^ Notably, none of the reported LFA cases were linked to myasthenia gravis.

**Table 2:** Summary of case reports of LFA.

Author	Year	Age/Sex	Site	Size (cm)	Clinical presentation	IHC	Treatment	Follow-up (Mo)/Status	Associations
Kuo et al[6]	2001	62M	AM	NA	Dizziness, dyspnea	Epithelial: AE1(+), CK14(+), CK19(+).	Thymectomy	80/ANED	B1 thymoma, PRCA
Aydin et al [7]	2012	23F	AM	21 × 7 and 5 × 7	Chest pain, dyspnea	NA	Total resection	12/ANED	
Qu et al[8]	2013	21M	AM	10 × 6 × 4	No symptom	Epithelial cells: AE1/AE3(+), CK19(+). Lymphocytes: CD3(+), CD20(+).	Thymectomy	46/ANED	
Makdisi et al[9]	2015	20M	AM	23 × 14 × 5	Fever, cough, night sweats	NA	Total resection	6/ANED	
Hui et al[10]	2018	29M	AM	5.4 × 2.4 × 6.5	Cough, expectoration	Epithelial cells: CK19(+). Lymphocytes: CD3(+), CD20(+), TdT(-).	Total resection	NA/ANED	with B1 thymoma
Kurebayashi et al[11]	2021	55F	AM	4.5 × 1.8 × 1.3	No symptom	Epithelial cells: CK19(+). Lymphocytes: CD3(+), CD20(+), TdT(only a few +).	Thymectomy	NA	
Hakiri et al [12]	2021	28M	AM	8.8 × 6.7 × 4.2	No symptom	Epithelial cells: AE1/AE3(+). Lymphocytes: CD3(+), TdT(+).	Total resection	6/ANED	
Bolca et al [13]	2021	64F	AM	16 × 8 × 6	Dyspnea	NA	Total resection	48/ANED	
Den Bakker et al[14]	2022	17M	AM	12.5 × 8.0 × 2.5	Incidental finding in work-up for pneumonia	Epithelial cells: AE1/AE3(+), p63(+). Lymphocytes: CD3(+), TdT(+),CD20(+).	Thymectomy	ANED	
Fu et al[5]	2022	21M	AM	9.2 × 5 × 2.1	Fever	Epithelial cells: AE1/AE3(+),CK19(+). Lymphocytes: CD3(+), CD20(+).	Total resection	48/ANED	
Yang et al [15]	2023	59F	AM	3.9 × 1.8	Chest tightness	Epithelial cells: CK19(+), P63(+), EMA(+). Lymphocytes:CD3(+), CD20(+).	Thymectomy	19/ANED	
		33F	AM	7.1 × 7.5	No symptom	Epithelial cells: CK19(+), P63(+), EMA(+). Lymphocytes:CD3(+), CD20(+).	Thymectomy	7/ANED	
		37M	AM	9.9 × 9.8 × 4.9	Dyspnea	Epithelial cells: CK19(+), P63(+), EMA(+). Lymphocytes: CD3(+), CD20(+).	Total resection	30/ANED	
Kazemi et al [16]	2024	30F	AM	10.5 × 7.5 × 5.0	No symptom	NA	Total resection	ANED	

AE = cytokeratin antibody, AM = anterior mediastinum, ANED = alive with no evidence of disease, CD = cluster of differentiation, cm = centimetre, CK = cytokeratin, EMA = , F = female, IHC = immunohistochemistry, LFA = lipofibroadenoma, M =male, NA = not available, PRCA = pure red cell aplasia, TdT = terminal deoxynucleotidyl transferase.

Histologically, all reported cases of LFA consistently exhibit an epithelial component composed of small, bland cells with sparse cytoplasm. These cells are arranged in interconnected branching strands that traverse a background of fibrous connective tissue and mature adipose tissue. The branching architecture of the epithelial strands is somewhat reminiscent of mammary fibroadenoma. They are intimately associated with both the adipocytic component and the paucicellular stromal tissue. Notably, features indicative of malignancy (such as cytologic atypia, significant proliferative activity, or necrosis) have not been described in LFA.

Scattered lymphocytes are present within the lesion. In contrast to other thymomas, a defining feature of this lesion is the uniform absence or only minimal presence of TdT-positive immature T lymphocytes.^[11,12]^ Additionally, calcification may be present in a minority of cases.^[10,12]^

By immunohistochemistry, the epithelial cells are positive for CK19, cytokeratin antibody 1/cytokeratin antibody 3, epithelial membrane antigen, and P63, and the lymphocytes express CD3 and CD20.

The histogenesis of LFA remains controversial. One hypothesis suggests a perivascular origin, based on observed morphological transitions between vascular-wall elements (smooth muscle cells and pericytes) and the tumor’s fibroblastic and immature adipocytic components.^[11]^

However, no such morphological transition was observed in our case. In contrast, other researchers propose that LFA may arise within native thymic tissue.^[12]^ A fundamental question regarding its nature remains unresolved: although the current (fifth) edition of the WHO Classification of Thoracic Tumors lists LFA under “thymic epithelial tumors” and designates it as “benign,” it is still unclear whether LFA represents a true clonal neoplasm or a hamartomatous malformation. This uncertainty is compounded by its frequent association with adjacent type B1 thymoma, which has led to the hypothesis that thymic epithelial cell precursors may give rise to these lesions.^[1]^ In support of a nonneoplastic origin, Kurebayashi et al.^[11]^ suggested that the epithelial components may not be neoplastic, highlighting morphological continuity between the slender epithelial bands in LFA and the nonneoplastic epithelium in hyperplastic thymic tissue: a histological feature that was also observed in our case. Further supporting this view, a recent molecular study of an LFA case detected no recurrent somatic mutations or characteristic structural variants.^[14]^

The present case exhibits a disorganized mixture of histologically benign thymic epithelial elements, mature adipose tissue, and fibrous connective tissue in varying proportions. The novel finding is a lesion characterized by lobular hyperplastic epithelium with ductal differentiation admixed with areas of classic LFA morphology. Given these features, the lesion did not correspond completely to any established category of thymic tumor. This composite histology bears a striking resemblance to hamartomas seen in other organs, particularly the breast. Consequently, we hypothesize that this lesion is best classified as a thymic hamartoma and further speculate that previously reported LFAs might also represent a form of hamartoma rather than a true epithelial neoplasm.

The lesion must be distinguished from several other benign and malignant mediastinal tumors, particularly those containing adipocytic or spindle cell components, such as thymolipoma, thymofibrolipoma, sclerosing thymoma, and teratoma. Thymolipoma is an uncommon thymic neoplasm that has been associated with severe conditions such as myasthenia gravis and autoimmune disorders. It is characterized by a predominant proliferation of mature adipose tissue interspersed with islands of benign thymic tissue containing lymphocytes and Hassall corpuscles. It lacks the prominent epithelial strands and fibrous tissue component, which is a key distinguishing feature. Thymofibrolipoma has been described as a variant of thymolipoma, characterized by extensive areas of collagenous tissue interspersed with islands of mature adipose tissue and strands of thymic tissue. Some researchers suggest that, based on previous descriptions of the histopathologic features and microscopic images of LFA and thymofibrolipoma, these 2 terms likely refer to the same pathologic entity.^[17]^ Sclerosing thymoma, also referred to as sclerosed or degenerative thymoma, is characterized by extensive hyalinization and fibrous sclerosis of the stroma. Within this sclerotic background, cords of epithelial cells are embedded, and the lesion lacks admixed adipose tissue. It is frequently associated with coagulative necrosis, dystrophic calcification, and cholesterol granulomas. According to the latest WHO classification, sclerosing thymoma is regarded as a sclerotic variant arising within other conventional types of thymoma and has therefore been removed as a distinct entity from the classification.^[1]^ Both mature and immature teratomas contain well-differentiated tissues derived from 2 or more germ cell layers (ectoderm, mesoderm, endoderm). Mature teratomas may contain elements such as skin, hair, teeth, or bone. Immature teratomas feature primitive, fetal-type tissues, most commonly neuroectoderm.

In summary, our review of the 14 previously reported LFA cases confirms their histologic uniformity. Clinical follow-up (the longest exceeding 80 months) after complete surgical excision has been universally favorable across all cases, with no reported recurrences, indicating that complete surgical resection is curative and represents the treatment of choice. Based on the features of our case and the existing literature, we propose that LFA may be more accurately conceptualized as a hamartomatous lesion of the thymus. Current understanding of this entity remains confined to the histopathologic level; accrual of more cases with comprehensive molecular analysis is imperative to determine whether specific genetic alterations underlie its development.

## Author contributions

**Conceptualization:** Ning Zhou, Fanrong Wang.

**Data curation:** Shuya Hu, Liqiao Chen.

**Writing – original draft:** Ning Zhou, Shuya Hu.

**Writing – review & editing:** Liqiao Chen, Hegang Wu, Fanrong Wang.
